# Molecular pathways of oestrogen receptors and β‐adrenergic receptors in cardiac cells: Recognition of their similarities, interactions and therapeutic value

**DOI:** 10.1111/apha.12978

**Published:** 2017-10-30

**Authors:** J.O. Machuki, H.Y. Zhang, S.E. Harding, H. Sun

**Affiliations:** ^1^ Department of Physiology Xuzhou Medical University Xuzhou China; ^2^ National Heart and Lung Institute Imperial College London UK

**Keywords:** β‐adrenergic receptors, cardioprotection, crosstalk, GPR30, intracellular signalling, oestrogen receptors

## Abstract

Oestrogen receptors (ERs) and β‐adrenergic receptors (βARs) play important roles in the cardiovascular system. Moreover, these receptors are expressed in cardiac myocytes and vascular tissues. Numerous experimental observations support the hypothesis that similarities and interactions exist between the signalling pathways of ERs (ERα, ERβ and GPR30) and βARs (β_1_AR, β_2_AR and β_3_AR). The recently discovered oestrogen receptor GPR30 shares structural features with the βARs, and this forms the basis for the interactions and functional overlap. GPR30 possesses protein kinase A (PKA) phosphorylation sites and PDZ binding motifs and interacts with A‐kinase anchoring protein 5 (AKAP5), all of which enable its interaction with the βAR pathways. The interactions between ERs and βARs occur downstream of the G‐protein‐coupled receptor, through the G_αs_ and G_αi_ proteins. This review presents an up‐to‐date description of ERs and βARs and demonstrates functional synergism and interactions among these receptors in cardiac cells. We explore their signalling cascades and the mechanisms that orchestrate their interactions and propose new perspectives on the signalling patterns for the GPR30 based on its structural resemblance to the βARs. In addition, we explore the relevance of these interactions to cell physiology, drugs (especially β‐blockers and calcium channel blockers) and cardioprotection. Furthermore, a receptor‐independent mechanism for oestrogen and its influence on the expression of βARs and calcium‐handling proteins are discussed. Finally, we highlight promising therapeutic avenues that can be derived from the shared pathways, especially the phosphatidylinositol‐3‐OH kinase (PI3K/Akt) pathway.

## 1 INTRODUCTION

The risk of cardiovascular diseases (CVDs) is higher in aged women compared to that of pre‐menopausal women.^1^ In addition, development of CVDs in men occurs at a relatively young age, while the risk of CVDs in women accelerates after menopause.^1^ These observations are attributed, in part, to gender‐related cardioprotective roles of oestrogen. Moreover, numerous studies have described the expression of oestrogen receptors (ERs) in various tissues of the cardiovascular system (CVS). There are 3 classes of ERs: the ERα, ERβ and the G‐protein‐coupled receptor 30 (GPR30). All these ERs are expressed in the heart cells and in the vascular vessels.^2^, ^3^, ^4^, ^5^ Each receptor subtype shows variation in function and in tissue‐specific expression. Based on ligand specificity, oestrogen and some of its metabolic intermediate products activate the ERs triggering both genomic and non‐genomic actions.^6^ Results from several experiments have implicated oestrogen in the chronotropic and inotropic functions of the heart,^7^, ^8^, ^9^ and in cardiac perfusion.^10^, ^11^, ^12^

In their recent study, Debortoli et al.^10^ showed that activation of GPR30 modulated coronary circulation by regulating coronary perfusion pressure in rats. Accordingly, left ventricular diastolic dysfunction is predominant in post‐menopausal women.^13^ Consistent with these reports, Giraud et al.^14^ used Magness et al.'s menopause model^15^ and showed that left ventricle diameters and end‐diastolic volume were elevated by chronic oestrogen replacement in this model. In the ovine model, 3 research groups showed that 17β‐oestradiol administration increased coronary blood flow significantly.^15^, ^16^, ^17^, ^18^, ^19^, ^20^ Collectively, they showed that the pattern of rises in coronary perfusion is independent of patterns of rises in cardiac output. They also noted that the pattern of raises in cardiac output was graded, that is a 30‐ to 60‐min delay followed by an increase and a plateau at 90‐120 minutes, a phenomenon observed in reproductive tissues such as uterus and mammary gland.^15^, ^20^ Mershon et al. confirmed that these effects of oestrogen are ER‐dependent as they were prevented by pre‐treatment with antagonist ICI‐182 780.^18^

**Figure 1:**
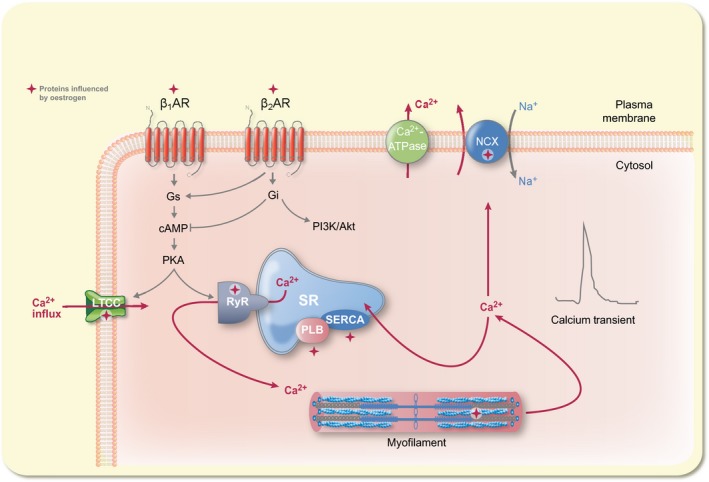
Cardiac Ca^2+^‐handling proteins and Ca^2+^ trafficking in cardiomyocyte regulated by βARs and oestrogen. Illustration of a network of calcium‐handling proteins and Ca^2+^ trafficking in cardiomyocyte which are activated and regulated by βARs and oestrogen. Purple arrows indicate movement of Ca^2+^. The symbol 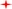 indicates points at which oestrogen exerts influence on cardiac contractile function. Abbreviations: LTCC, L‐type channel; RyR, ryanodine receptor; SR, sarcoplasmic reticulum; SERCA, sarcoplasmic reticulum Ca^2+^‐ATPase; NCX, Na^+^/Ca^2+^ exchanger pump; PLB, phospholamban

The heart rhythm and contraction are mainly regulated by the sympathetic nervous system (SNS), via the β‐adrenergic receptors (βARs) that connect and convey the SNS signals to the heart. βARs are divided into β_1_AR, β_2_AR and β_3_AR.^21^ Interestingly, signalling pathways of ERs are intertwined with those of the βARs pointing to the possibility of functional convergence in modulating the physiology of the CVS. In fact, crosstalk between ERα and α_1b_‐adrenergic receptor was reported previously.^22^, ^23^ Furthermore, we and others established that oestrogen alters gene expression of βARs and calcium (Ca^2+^)‐handling proteins of the CVS.^24^, ^25^, ^26^ Proteins that regulate cardiac Ca^2+^ include the Na^+^/Ca^2+^ exchanger pump (NCX), L‐type Ca^2+^ channel (LTCC), phospholamban (PLB), sarcoplasmic reticulum Ca^2+^‐ATPase (SERCA) and ryanodine receptors (RyRs; Figure 1).^27^ Consequently, the effects of oestrogen on the Ca^2+^‐handling proteins have direct implications on the contractile machinery of the myocardium.

In recent decades, the need to understand the cardiovascular functions of oestrogen and βARs has received much interest from researchers. The discovery of GPR30,^28^ which shares structural features with βARs, has expanded the functional scope of oestrogen. In this regard, it is important to re‐evaluate the relationships between ER and βAR signalling pathways and their interdependence in modulating the cardiovascular physiology. This review focuses on the recent experimental studies to describe the roles and mechanisms of ERs and βARs. We firstly discuss their classification, functions and the basis of cardiac physiology. We then provide novel illustrations on the points of integration between oestrogen and adrenergic signalling pathways. Our aim is to provide evidence for the hypothesis that there are interactions and functional cooperation between ER and βAR signalling pathways, particularly in the heart. We highlight the therapeutic potential of the interactions and explore their implications on the postulated cardioprotection conferred by oestrogen, β‐blockers and Ca^2+^ channel blockers. We also discuss the ERs and βARs as coregulators of cardiac Ca^2+^‐handling proteins.

## 2 A RECAP OF β‐ADRENERGIC RECEPTORS IN THE CARDIOVASCULAR SYSTEM

### 2.1 βAR‐specific features

βARs are members of the G‐protein‐coupled receptors (GPCRs) that classically form 7 transmembrane loops, with extracellular and intracellular terminals. Three βAR subdivisions, β_1_AR, β_2_AR and β_3_AR, are encoded by different genes.^21^ Moreover, the 3 receptors are expressed in the plasma membrane in the CVS,^29^ as well as in the cardiac nuclear envelope of adult rats and mouse myocytes for β_1_AR and β_3_AR.^30^, ^31^, ^32^In heart myocytes, the number of β_1_ARs is higher than that of β_2_ARs, while β_3_ARs show the least abundance.^33^ βARs are linked to heterogeneous intracellular signalling pathways and proteins. In addition, their expressions vary under physiological and pathological conditions.^34^

### 2.2 βAR‐specific signalling

βARs are activated by noradrenaline and adrenaline released from the SNS and adrenal glands respectively. However, once activated, the βARs trigger diverse intracellular pathways.^35^ β_1_ARs couple to the stimulatory unit of the G protein (G_αs_) leading to the synthesis of cyclic adenosine monophosphate (cAMP) by adenylyl cyclase (AC) enzyme. On the other hand, β_2_ARs are pleiotropic receptors that couple to the G_αs_, the inhibitory G protein (G_αi_) and the G_βγ_.^36^, ^37^ At the physiological state, β_2_ARs couple to the G_αs_, whereas at high adrenaline concentration, they switch to G_αi_, a phenomenon referred to as stimulus‐mediated trafficking.^38^, ^39^ Activation of the β_2_AR/G_αi_ pathway inhibits cAMP production, an opposing effect to β_2_AR/G_αs_ and β_1_AR/G_αs_ activation (Figure 1).

With regard to structure and function, the β_3_ARs display distinct differences to β_1_ARs and β_2_ARs. Notably, the cytoplasmic C‐terminus of the β_3_ARs lacks the target amino acid sequences for protein kinase A (PKA) and cardiac G‐protein‐coupled receptor kinase 2 (GRK2) phosphorylation.^40^, ^41^ Consequently, β_3_ARs are less susceptible to PKA/GRK2‐mediated receptor recycling and desensitization in response to hyperstimulation.^40^ Two isoforms, β_3a_AR and β_3b_AR, were reported in Chinese hamster ovary (CHO) cells and 3T3‐L1 adipocytes.^42^, ^43^, ^44^, ^45^, ^46^ The β_3a_AR isoform coupled to the G_αi_, whereas β_3b_AR coupled to both G_αs_ and G_αi_. At present, there are no reports regarding the existence of the 2 isoforms in human cardiac cells. β_3_AR has largely been associated with metabolic functions. Nevertheless, β_3_AR stimulation induced positive inotropy in human atrial cells,^47^ but it had no effect on the inotropy of human ventricular cells.^48^ β_3_AR may influence chronotropic functions through the nitric oxide (NO)/guanosine 3′,5′‐monophosphate (cGMP) pathway.^49^ Activation of plasma β_3_AR‐NO synthase/guanylyl cyclase pathway was shown to influence the nuclear β_3_AR‐mediated gene transcription, suggesting a crosstalk between the surface and nuclear β_3_ARs.^50^

### 2.3 The basis of the heart's function

βARs mediate the SNS regulation of the cardiac functions.^51^ These functions are primarily orchestrated by activation of β_1_ARs, which constitute up to 80% of the entire cardiac βAR density of healthy human, and to a lesser extent by the β_2_ARs.^33^, ^35^ Moreover, β_2_ARs have a higher affinity for adrenaline, while β_1_ARs have almost equal affinities for both noradrenaline and adrenaline.^39^ On the other hand, activation of the β_3_ARs is largely associated with negative inotropy during catecholaminergic stress.^47^

Once activated, βARs initiate cAMP synthesis by coupling to the G_αs_, a GTP‐binding protein. This cAMP, in turn, activates PKA. What follows is the induction of intracellular rise in Ca^2+^ transients via the tightly regulated network of ion channels. PKA‐mediated inotropic effects are orchestrated through the phosphorylation of 2 main channels: the LTCC located in the T‐tubular network formed by sarcolemmal membrane invaginations and the RyR2 receptors on the SR membrane. Phosphorylation of LTCC allows Ca^2+^ entry as inward current.^52^ These Ca^2+^ currents further stimulate Ca^2+^ release from SR, the intracellular stores, by the opening of RyR2 receptors which are also phosphorylated by PKA. This phenomenon is referred to as calcium‐induced calcium release. The resultant Ca^2+^ transient activates the myofilament protein troponin C turning on cardiomyocyte contraction. The size of Ca^2+^ transients is a key determinant of the strength of the contraction.^27^ PKA also regulates cardiac relaxation by phosphorylating phospholamban (PLB), a modulator of SERCA. In its unphosphorylated state, PLB inactivates SERCA. This effect is reversed following PLB phosphorylation which permits Ca^2+^ uptake back to the SR by SERCA. In addition, sarcolemmal Na^+^/Ca^2+^ exchanger pump (NCX) accelerates Ca^2+^ extrusion, which together with SR Ca^2+^ uptake diminishes the Ca^2+^ transient resulting in relaxation.

Besides the classical cAMP/PKA pathway, cAMP also acts through the recently described intracellular protein named exchange protein directly activated by cAMP (EPAC).^53^ Classified into EPAC1 and EPAC2, these proteins bind cAMP and function as guanine exchange factors (GEFs) for Ras superfamily. The EPAC pathway amplifies the cardiovascular functions of β_1_AR/cAMP and provides alternative modulation of βAR activation. Indeed, both EPAC1 and EPAC2 are present in cardiomyocytes.^53^ Diverse physiological roles of EPAC proteins have been recently reviewed by Lezoualc'h et al.^54^ Activation of EPAC was linked to ventricular hypertrophy, vasorelaxation and in the regulation of Ca^2+^ through RyR and PLB phosphorylation,^54^ indicating synergism between the cAMP/PKA and cAMP/EPAC pathways.

Another downstream target of β_1_AR activation is the multimeric protein Ca^2+^/calmodulin kinase II (CaMKII).^55^ Activation of this kinase indirectly relies on the PKA‐mediated rise in cytosolic Ca^2+^ and intracellular levels of calmodulin.^56^ Recent findings reveal that CaMKII activation augments the LTCC current and increases the RyR open probability^57^ and phosphorylation of PLB,^58^ showing its participation in cardiac contractility. CaMKII has also been associated with detrimental effects including apoptosis, necroptosis and arrhythmias.^59^

## 3 CLASSIFICATION, LOCALIZATION AND DISTRIBUTION OF ERS IN THE CVS

The cardiovascular functions of oestrogen are mediated by ERs. These cellular receptors are categorized as nuclear receptors (ERα and ERβ), which modulate transcription of specific gene sets, and membrane‐bound receptor (GPR30, also known as GPER1), which mediates rapid, non‐genomic actions of oestrogen. ERs are expressed in cardiomyocytes,^3^ cardiac fibroblasts^4^ and VSMCs;^5^ however, their expression and cellular locations are not fully understood. For instance, Pugach et al.^3^ reported that ERβ was not expressed in either neonatal or adult male or female mouse or rat ventricular myocytes. This observation is inconsistent with earlier reports.^60^, ^61^, ^62^ In addition, there are controversies surrounding the cellular localization of GPR30. In particular, some researchers reported that GPR30 was nearly confined to the endoplasmic reticulum in COS cell lines,^63^ while others observed both cytosolic and membrane localization in HEK293 cells^64^ and in rat VSMCs.^65^ The differences in these reports may be related to tissue‐specific variations. It is also important to note that oestrogen is a lipophilic hormone that crosses the plasma membrane to access the intracellular receptors. Therefore, both membrane and subcellular localization of GPR30, as observed, are conceivable as they are accessible to oestrogen.

With regard to gender, a study carried out on VSMCs of rats showed that GPR30 expression was similar in both males and females.^65^ However, gender differences with regard to ERα and ERβ expression were also reported. Whereas the mRNA levels of ERα were equivalent in hearts of both men and women,^3^, ^66^ ERβ had greater expression in males than females in both healthy and diseased human hearts.^67^ However, these observations are in contradiction to another report that showed an opposite expression pattern where ERβ expression was not different in male and female cardiomyocytes, while ERα expression varied with gender.^68^ Elsewhere, ERα and ERβ protein levels in male and female rabbit hearts were not different.^69^ Further studies are advocated to reconcile these findings.

In addition, Ma et al.^65^ observed that subcellular location of ERα was not influenced by its activation; a similar observation was reported for GPR30 in a subsequent study.^64^ However, change in subcellular location of the ERs may vary as in heart failure. In healthy hearts, ERα was localized to the intercalated disc, while in failing hearts, its location shifted away from the intercalated discs.^66^ This implies that cardiomyopathies may influence the subcellular localization and by extension the signalling of the ERs. Moreover, oestradiol supplementation in ovariectomized (OVX) rats increased ERα and ERβ protein levels.^70^ Variation in relative abundance of the ERs was recently reported. Quantitative real‐time PCR analysis of male mouse ventricle found that GPR30 mRNA levels were thrice those of ERα and 17‐fold greater than those of ERβ.^62^ Different genes located on different chromosomes encode each ER subtype. While alternative splicing of the gene transcripts leads to multiple subtypes of ERα and up to 5 described transcripts of ERβ,^71^ GPR30 only exists in 1 isoform.^3^, ^6^, ^72^ The distinct features of the ER subtypes are outlined in Table 1. Taken together, expression of the ER subtypes in the cardiovascular system remains contentious with regard to tissue‐specific expression. Discrepancies from the previous reports could be due to the methods used or species of tissue investigated. Further investigations are required to resolve the inconsistencies.

**Table 1:** Features and classification of oestrogen receptors

Receptor features	GPR30	ERα	ERβ
Cellular location	Plasma membrane^96^	Nucleus^83^, ^169^	Nucleus^130^
	Cytosol^64^	Cytosol^60^, ^130^	Cytosol^60^
		Plasma membrane^130^, ^170^	Plasma membrane^82^, ^169^
Onset of physiological effects	Rapid actions (effects within seconds to minutes)^102^	Rapid and genomic action (effects within minutes to days)^82^, ^102^, ^171^, ^172^, ^173^, ^174^	Rapid and genomic action (effects within minutes to days)^82^, ^83^, ^173^, ^174^, ^175^
Genetics	GPER gene located on chromosome 7p22.3^72^	ESR1 gene located on chromosome 6q25.1^176^	ESR2 gene located on chromosome 14q23.2^178^
	No introns, 1 isoform^72^	8 exons, 3 isoforms^176^	8 exons, 5 isoforms^178^, ^179^
	Protein size 375 amino acids^72^	Protein size 595 amino acids^177^	Protein size 530 amino acids^178^
Cardiovascular tissue distribution	Cardiac fibroblasts^4^	Cardiac fibroblasts^2^	Cardiac fibroblasts^2^
	Vascular tissues^180^	Vascular tissues^93^, ^102^	Vascular tissues^93^
	Cardiomyocytes^62^	Cardiomyocytes^2^, ^3^	Cardiomyocytes (unresolved)
Ligands	E2^62^	E2^130^	E2^83^
	G‐1^181^	PPT^76^	DPN^76^
Relative abundance in cardiac cells	Highest^62^	Low^62^	Lowest^62^

E2, 17β‐oestradiol; PPT, propylpyrazoletriol; DPN, propylpyrazoletriol; G‐1, GPR30 agonist; ERα, oestrogen receptor α; ERβ, oestrogen receptor β; GPR30, G‐protein‐coupled oestrogen receptor 30.

## 4 ER ACTIVATION, SIGNALLING PATHWAYS AND CELL FUNCTIONS

### 4.1 ER activation

Similar to other steroids hormones, oestrogen signalling is initiated by the binding of 17β‐oestradiol or xenoestrogens^73^ and oestradiol metabolites^74^, ^75^ to ER. Synthetic receptor‐specific agonists with selective binding affinities have also been developed: propylpyrazoletriol (PPT) for ERα, diarylpropionitrile (DPN) for ERβ and G1 for GPR30.^76^ Noteworthy, each receptor subtype or isoform displays different affinities to 17β‐oestradiol and other oestrogenic ligands.^77^, ^78^ Moreover, oestrogens are of different forms (estrone, oestradiol and estriol) which exist in a dynamic equilibrium in circulation. Considering that oestrogen activates multiple receptors, ER subtype–specific functions determine cellular responses to oestrogen stimulation. It has been postulated that the balance between oestrogen forms is responsible for activation of different signalling pathways under certain physiological conditions based on the premise that ERs possess different affinities for each oestrogen subtype.^78^ Furthermore, 17β‐oestradiol synthesis occurs through enzymatic modifications of precursors such as androgens by aromatase enzyme. Considering that aromatase is expressed within the heart,^68^ the possibility of cardiac oestrogen synthesis further augments the importance of oestrogen to the cardiovascular physiology in addition to circulating oestrogens. It is also likely that the adipose tissue surrounding the heart is the source of the C19 androgen conversion to C18 oestrogen, considering that epicardial fat covers up to 80% of heart's surface and constitutes 20% of heart's weight.^79^

### 4.2 Signalling pathways

#### 4.2.1 Receptor‐mediated signalling: genomic vs non‐genomic pathways

Binding of oestrogen to its receptors (membrane or nuclear) triggers 2 types of cellular effects defined by the timing of onset. (i) Part of the effects occurs through the well‐established pathway of ER‐mediated transcription of certain genes. Conventionally, this pathway is known as a genomic pathway and occurs within hours to days.^80^ The ERα and ERβ receptors largely execute these genomic functions. Upon oestrogen binding, these ERs undergo conformational changes allowing nuclear translocation and dimerization of the oestrogen‐ER complex with oestrogen response elements, found at promoter areas of specific genes. Through this mechanism, oestrogen influences expression of cellular proteins. However, this pathway is not exclusive to nuclear receptors. Activation of membrane receptor GPR30 induced gene transcription.^4^ (ii) Another pathway that emerges after oestrogen binding is the non‐genomic pathway. This pathway requires activation of several different signalling cascades that alter cellular functions of proteins and ion channels. Most of these actions occur within seconds or minutes and are regulated by ERα and GPR30.^7^, ^62^, ^81^ There are reports indicating the presence of ERβ in the cytosol and plasma membrane and that they are responsible for rapid non‐genomic signalling in endothelial cells. It is yet to be established whether ERβ exists on the plasma membrane of adult cardiomyocytes.^60^, ^82^, ^83^, ^84^ In addition, crosstalk between membrane ERs and nuclear ERs has been reported.^85^ There is growing interest to decipher mechanisms that underlie non‐genomic oestrogen signalling. How the non‐genomic oestrogen pathways integrate with βAR pathways forms the basis of the discussion dealt with in Section 3 of this article. Moreover, the interaction of ER signalling with adrenergic receptor pathways was observed between the ERα and α_1b_‐adrenergic receptors.^22^

#### 4.2.2 Receptor‐independent signalling

Besides the conventional receptor‐mediated mechanisms of oestrogen, experimental observations have hinted at the possibility of an alternative mechanism that does not involve membranous or nuclear ERs.^81^, ^86^, ^87^ This mechanism falls in the category of rapid and non‐genomic pathways and does not involve oestrogen‐receptor binding. A previous experiment showed that oestrogen induced negative inotropy in ERα and ERβ knockout mouse cardiomyocytes and its inhibition of the LTCC current was not altered from wild‐type myocytes.^88^ The same laboratory later demonstrated that oestrogen directly interacts with the LTCC protein and inhibits LTCC current even at resting state on cultured HEK293 cells.^86^ Indeed, similar observations have been reported for a broad range of ion channels (see review^89^). Research on the rapid non‐genomic roles of oestrogen has been primarily focused on the membranous receptors. Therefore, the observation that oestrogen could bind directly to ion channels introduces a reclassification of its mechanism of actions and adds to the growing debate on its non‐genomic functions.

### 4.3 Tissue‐specific functions

Oestrogen plays several functions in the CVS. Here, we highlight some of the cell‐specific roles of oestrogen without much detail because of the limitation defined by the purpose of this review. Activation of GPR30 inhibited proliferation of rat cardiac fibroblasts and collagen synthesis in both *in vivo* and *ev vivo* settings.^4^ These effects were attributed to oestrogen‐induced expression of cell cycle proteins and alterations in expression of matrix metalloproteinase‐12. GPR30 also mediates cardioprotection against ischaemia/reperfusion injury by improving the heart function, reducing infarct size, and mitochondrial Ca^2+^ overload.^62^ On the other hand, ERα agonists induced vasodilation on vascular smooth muscle cells of the aorta.^90^ In our previous study, we demonstrated that oestrogen and G1 decreased the expression of β_1_ARs and induced negative inotropy.^24^, ^91^ ERβ has also been reported to offer cardioprotection in cardiomyocytes.^92^ Together, these findings demonstrate that ERs are important effectors of oestrogen signals in cardiovascular tissues.

## 5 SIMILARITY IN MOLECULAR PATHWAYS OF ERS AND ΒARS

The crosstalk between ERs and βARs is a concept that was revealed from earlier studies.^22^, ^23^ Evidence from recent studies further recognizes oestrogen as a key hormone that influences the expression of βARs^26^, ^91^ and cardiac ion‐handling proteins.^93^, ^94^ Moreover, oestrogen regulates the cardiac contractile functions, which are otherwise under the control of adrenergic receptors (details discussed in Sections 4 and 4.1). Intriguingly, the structure (of GPR30) and signalling pathways of ERs are functionally closer/related to those of the βARs, at least partially.^72^, ^93^, ^95^, ^96^, ^97^, ^98^ Some of the cellular roles of ERs synergize or oppose the effects produced by βAR activation. Therefore, here we explore, side by side, the correlation among β_1_ARs, β_2_ARs and β_3_ARs vs. ERα, ERβ and GPR30. We discuss various points of integration between their signalling pathways. We note 3 main pathways along which these classes of receptors interact.

### 5.1 Signalling along the GPCR/G_αs_/cAMP pathway

**Figure 2:**
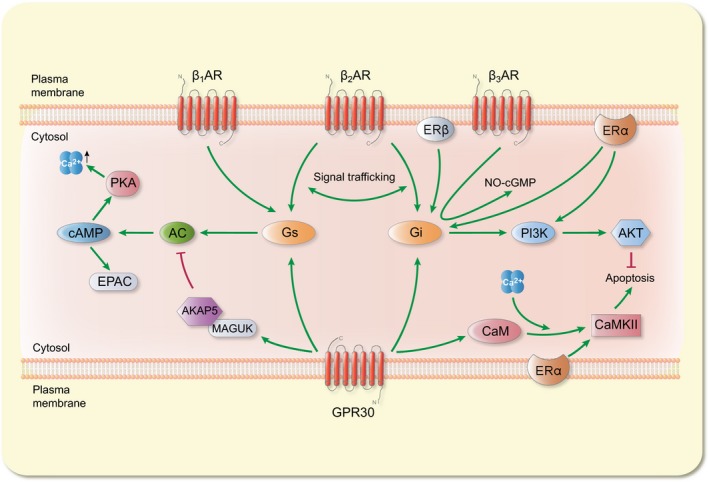
Interaction of oestrogen signalling and beta‐adrenergic signalling pathways. The symbol 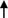 represents cytosolic Ca^2+^ rise. The symbol 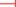 represents inhibition signal. The symbol 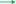 represents activation signal. Signalling pathways of βARs (β_1_AR, β_2_AR and β_3_AR) and ERs (ERα, ERβ and GPR30) are integrated through the G_s_ and G_i_ pathways. Effector proteins PKA and EPAC affect the G_s_‐cAMP signals. The resultant effects play crucial roles in cardiac contraction by increasing cytosolic Ca^2+^ levels. Alternatively, the receptors may activate the G_i_, which mediates anti‐apoptosis signals through the PI3K/Akt pathway. Elevated cytosolic Ca^2+^ levels activate CaM and CaMKII, which induces apoptosis. GPR30 may inhibit the AC enzyme through the MAGUK/AKAP5 complex. GPR30: G‐protein‐coupled receptor 30; E2: 17β‐oestradiol; EPAC: exchange protein directly activated by cAMP; AKAP5: A‐kinase anchoring protein 5; MAGUK: membrane‐associated guanylate kinase; CaM: calmodulin; CaMKII: Ca^2+^/calmodulin kinase II

Similar to βARs, GPR30 possesses PKA phosphorylation sites and PDZ binding motifs and associates with A‐kinase anchoring proteins (AKAPs).^64^ In addition, GPR30 uniquely possesses 4 CaMKII binding sites unlike all other GPCRs.^99^ Furthermore, like the βARs, GPR30 couples to the classical GPCR proteins, G_αs_ 100, 101 and G_αi_/_o_,^63^, ^96^, ^102^ in cardiovascular tissues (Figure 2). On this basis, activation of GPR30 partially mimics the signalling pathway of βARs with regard to its downstream cascades. Initial activation of β_1_AR, β_2_AR and GPR30 leads to coupling to G_αs_ protein.^96^, ^103^ Activation of G_αs_ triggers the production of cAMP by AC enzyme. Subsequently, PKA and EPAC amplify the cAMP signal. In coronary arteries, GPR30 was shown to activate this pathway including the production of PKA and EPAC proteins.^101^ The cAMP is hydrolysed by phosphodiesterases (PDEs) that determine the specificity of its signalling so as to avoid “off‐target” reactions through the creation of microdomains.^104^ This process occurs through the multimeric units formed between βAR/G_αs_/AC and PDEs.^104^ In addition, it is known that PKA interacts with PDE4 and facilitates the degradation of cAMP by associating with the scaffold proteins AKAPs.^105^ Therefore, considering the observation that GPR30 signals through the G_αs_/AC/cAMP pathway, it would be interesting to define whether the GPR30/G_αs_/AC complex also participates in compartmentalization of cAMP signals in cardiac cells. The PKA generated downstream of GPR30 might interact with PDEs, under the direction of AKAP5, to regulate cAMP degradation as for β_1_ARs and β_2_ARs. Moreover, we speculate that GPR30's ability to activate EPAC might also influence β_1_AR/cAMP/EPAC‐mediated functions. Besides GPR30, activation of the ERα elevated PKA in VSMCs of aortic tissue further illustrating oestrogen involvement in the GPCR/G_αs_/cAMP signalling pathway.^102^

Additional interactions are possible due to the structural resemblance between GPR30 and βARs. βARs interact with cellular proteins through PDZ motifs located at their C‐terminus regions. The PDZ motifs give a bearing on the localization and signalling of βARs. Of note, β_1_AR, β_2_AR and GPR30 possess type I PDZ binding motifs: ‐ESKV, ‐DSLL and ‐SSAV respectively.^106^, ^107^ PDZ domains recognize and bind to specific amino acid sequences of their target proteins.^106^ For instance, the β_1_AR (‐ESKV) motif was shown to be a determinant factor for its coupling to G_αs_ and not G_αi_. Induced disruption of this motif permitted β_1_AR/G_αi_ coupling.^106^ On the other hand, β_2_AR (‐DSLL) motif plays a role in its coupling to G_αi_.^108^ In comparison, the GPR30 PDZ motif (‐SSAV) was implicated in recycling and translocation of GPR30 in HEK293 cells,^64^ but it is not known whether this motif may influence GPR30's ability to couple to G_αs_ or G_αi_. In addition to the PDZ motifs, β_2_AR phosphorylation by PKA influences its ability to bind G_αs_ or G_αi_.^39^ Although GPR30 coupling to G_αs_ or G_αi_ may be dependent on cell/tissue type, collectively, these observations raise the possibility that PKA phosphorylation or disruption of its PDZ motif would have implications on its coupling to G_αs_ and G_αi_ as is the case for β_1_ARs and β_2_ARs. This possibility calls for further inquiry to the conditions under which GPR30 couples to G_αi_ and G_αs_, and the proteins that interact with its PDZ motif.

One functional implication of this motif draws from the recent observation that GPR30 inhibited βAR‐mediated production of cAMP in response to isoproterenol stimulation in HEK293 cells.^64^ This inhibitory effect was dependent on a complex formed by GPR30, through its PDZ motif, with membrane‐associated guanylate kinases (MAGUKs) and AKAP5 (Figure 2).^64^ The primary role of AKAPs is to bind and regulate the subcellular location of PKA. Interestingly, the binding of AKAP5 to β_1_ARs facilitated its recycling by enhancing PKA phosphorylation of the receptor.^109^, ^110^, ^111^ Moreover, it is established that oestrogen regulates expression of β_1_ARs**.** Therefore, an association of GPR30 with AKAP5 and possibly other unidentified proteins could be a mechanism through which oestrogen participates in the regulation of β_1_AR density. Further research should be carried out to characterize the interactions of GPR30 with AKAPs and their implications on cellular functions.

Although β_1_AR, β_2_AR and GPR30 are capable of coupling to G_αs_, their cellular effects are not similar. For instance, activation of β_1_ARs,^112^ ERα and GPR30^113^ triggers production of calmodulin and activation of CaMKII, while β_2_ARs and ERβ do not. Importantly, the observation that GPR30 activates cAMP through G_αs_ pathway challenges previous findings that oestrogen induced negative inotropy at both cardiomyocyte and organ levels. Therefore, the effects of GPR30/G_αs_/cAMP pathway may not be identical to the classical βAR/G_αs_/cAMP pathway, especially in cardiomyocytes. However, as we reported, oestrogen may tilt the activation of β_2_AR/G_αs_ or β_2_AR/G_αi_ pathways in certain disease conditions, as in stress‐induced cardiomyopathy.^114^

### 5.2 Signalling along the GPCR/G_αi_/PI3K/Akt pathway

As mentioned earlier, GPR30, like β_2_ARs and β_3_ARs, couple to the G_αi_ subunit.^96^, ^102^, ^115^ Furthermore, GPR30 activates the phosphatidylinositol‐3‐OH kinase (PI3K)/Akt pathway resulting in inhibition of apoptosis through regulation of the Bcl‐2 family of proteins.^24^, ^102^, ^115^, ^116^ The GPR30/PI3K/Akt‐mediated cardioprotection against ischaemia/reperfusion injury in cardiomyocytes was orchestrated through upregulation of anti‐apoptosis Bcl‐2 protein and downregulation of pro‐apoptosis Bax protein.^116^ In accordance with our previous report, inhibition of β_2_ARs exposes cardiomyocytes to cell death in ischaemic conditions.^117^ The β_2_AR/G_αi_ pathway triggered anti‐apoptotic signals through the PI3K/Akt pathway.^118^ These findings present the evidence that PI3K/Akt cardioprotective pathway is shared by the GPR30 and β_2_ARs. In addition to the GPR30, oestrogen activates the ERα, which directly binds the p85 alpha regulatory component of the PI3K.^119^

In addition, PKA phosphorylation of the β_2_ARs enables switching of its coupling from G_αs_ to G_αi_ under extreme catecholamine stimulation,^39^ a phenomenon referred to as signal trafficking (Figure 1). In this context, β_2_ARs act as a switch that coordinates synthesis of cAMP and indicates cross‐communication between β_1_AR and β_2_AR signalling. Moreover, AKAP5 tethering of PKA allows it to phosphorylate β_2_ARs.^120^ Considering that GPR30 possesses PKA phosphorylation sites, we speculate that through AKAP5, PKA phosphorylation of GPR30 may influence its ability to activate G_αs_ or G_αi_. Although we appreciate that such signal trafficking is intricately complicated and may involve different mechanisms, further research is necessary to determine whether this phenomenon can be replicated in adult cardiomyocytes. Unlike β_2_ARs, the conditions under which GPR30 activates G_αs_ or G_αi_ are not clearly understood. Perhaps a possible hint as to when GPR30 activates G_αi_ comes from the observation that under stress conditions, both β_2_ARs and GPR30 activate G_αi_/PI3K/Akt pathway to confer cardioprotection.^116^, ^118^ However, we recognize that the requirements for GPR30 coupling to G_αs_ or G_αi_ in cardiomyocytes need further investigation.

Unlike β_1_ARs, β_2_ARs and GPR30, β_3_ARs lack PKA phosphorylation sites.^40^ Therefore, for β_3_ARs, there is a great deal of variability with respect to their ability to activate both G_αs_ and G_αi_. Some β_3_AR splice variants were shown to display dual coupling to G_αs_ and G_αi_ in other cell types, although not in cardiac cells.^121^ β_3_ARs act through G_αi_/_o_ to suppress contractility via induction of NO‐cGMP pathway under chronic catecholaminergic stimulation.^122^ In addition, β_3_AR/NO/cGMP pathway was enhanced in the presence of β_1_AR blocker, which was interpreted to be beneficial in chronic volume‐overloaded heart.^123^ Based on the evidence presented above, the ER and βAR signalling pathways function as interdependent networks/partners whose roles have profound effects on the cardiovascular system. In summary, G_αs_ and G_αi_ act as pivots around which both ER and βAR signalling pathways converge. β_2_AR and GPR30 signal through the G_αs_/AC/cAMP and G_αi_/PI3K/Akt pathways in the cardiovascular system. Similarly, both GPR30 and ERα signalling cascades interact through the PI3K/Akt pathway (Figure 1). PI3K/Akt acts as a focal pathway that unifies GPR30‐, ERα‐ and β_2_AR‐mediated cardioprotection. In general, the G_αs_/AC/cAMP and G_αi_/PI3K/Akt pathways seem to trigger opposing effects. For example, the cAMP produced by βAR stimulation was shown to inhibit the activity of Akt kinase indicating an inverse relationship between the 2 pathways.^124^ Lastly, the net cellular effects of the interactions between ERs and βARs might be dependent on the cell/tissue type.

### 5.3 Localization of ERs and βARs to the caveolae

**Figure 3:**
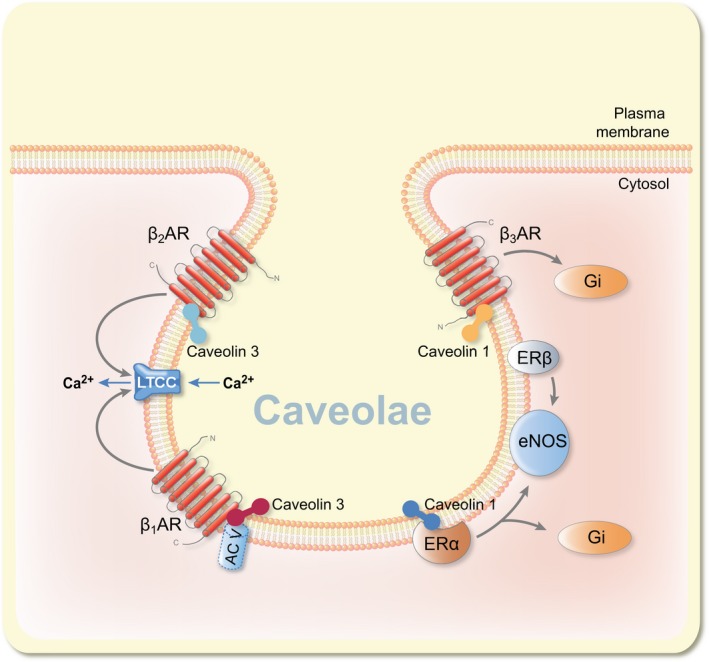
βAR and ER signalling through caveolae. In this view, β_2_AR associates with caveolin 3 and LTCC to transduce signals that increase cellular Ca^2+^. β_1_AR interacts with caveolin 3 and AC V to induce LTCC current. Caveolin 1 interacts with β_3_AR and governs its ability to couple to G_αi_. ERα colocalizes with caveolin 1 and signals through G_αi_ and activates eNOS pathway. ERβ mediates the activation of eNOS by oestrogen in the caveolae. AC V: adenylyl cyclase V; ERα: oestrogen receptor alpha; ERβ: oestrogen receptor beta; eNOS: endothelial nitric oxide synthase; LTCC: L‐type calcium channel

β_1_AR, β_2_AR and β_3_AR have been shown to signal and express in the caveolin‐rich fractions of the plasma membrane (Figure 3). Caveolin proteins are found in flask‐shaped subdomains of plasma membranes known as caveolae.^125^ β_2_AR localizes almost exclusively to caveolin 3‐rich membrane fractions of rat cardiomyocytes, while β_1_AR localizes to both caveolar and non‐caveolar membrane fractions.^126^ These spatial distributions play a role in the differential activation of cAMP signals by β_1_AR and β_2_AR.^127^ For instance, colocalization of β_2_AR with the Ca^2+^ channel LTCC and caveolin 3 is essential for its signalling and ability to invoke intracellular Ca^2+^,^128^ while caveolin 3 interaction with AC V acts as a scaffolding protein which participates in β_1_AR signals that induce LTCC current in ventricular cardiomyocytes.^129^ ERα was associated with eNOS activation in the caveolae of endothelial cells,^130^ while ERβ was found in the caveolae where it mediated eNOS signals.^82^ Strikingly, overexpression of β_2_AR enhanced vascular repair of endothelial progenitor cells in mice through the eNOS pathway.^131^ Therefore, localization of β_2_AR and ERβ in caveolae of vascular cells and their ability to signal through the eNOS pathway indicate functional cooperation between the 2 receptors. Caveolin 1 is a scaffold protein for both ERα and β_3_AR.^132^, ^133^ The association of β_3_AR with caveolin 1 was shown to govern its ability to couple to G_αi/o_ proteins in CHO‐K1 cells.^133^ On the other hand, it has been established that ERα and ERβ interact directly with G_αi_ and that these interactions occur in close proximity to the caveolae domains.^134^ The physiological relevance of the possible interactions among ERs, caveolins and βARs in adult cardiomyocytes remains to be fully established. Although there is a dearth of evidence regarding this view, the data sets reviewed here imply direct or indirect crosstalk among ERs and βARs. Further research is required to identify multilevel communications and interactions among these receptors.

A summary of the shared features is as follows:

- β_2_ARs, β_3_ARs, ERα, ERβ and GPR30 couple to G_αi_ subunit.
- β_1_ARs, GPR30 and ERα activate calmodulin/CaMKII.
- β_1_ARs, β_2_ARs and GPR30 couple to G_αs_ subunit.
- β_2_ARs, ERα and GPR30 trigger the PI3K/Akt pathway.
- β_3_AR and ERα associate with caveolin 1, while β_1_AR and β_2_AR associate with caveolin 3.

## 6 OESTROGEN INFLUENCE ON THE EXPRESSION OF βARS

Expression of βARs in the cardiovascular vessels is influenced, in part, by age, by gender and by drugs targeting these receptors.^135^ The ratio of β_1_ARs, β_2_ARs and β_3_ARs may also vary with disease status.^136^ In pre‐menopausal women, the cardiac expression of β_1_ARs and β_2_ARs decreases with age until menopause after which it stabilizes.^135^ On the contrary, there is no significant association between age and β_1_AR/β_2_AR expression in men.^135^ Together with other numerous animal experiments, these observations seem to indicate that sex hormones, particularly oestrogen, play regulatory roles in the expression of βARs. Moreover, these roles may be due to the direct action of oestrogen on βAR signalling cascades or indirectly through adaptative responses to oestrogen environment.

### 6.1 β_1_AR expression

Our studies^91^ and others^25^, ^137^, ^138^ systematically showed that ovariectomy (OVX) increased the expression of β_1_ARs and induced negative inotropy in rat hearts subjected to ischaemia/reperfusion injury (I/R) and, in addition, that this role of oestrogen was mediated by the ERα.^91^ On the other hand, activation of GPR30 in ventricular myocytes from OVX rats reversed the effects of OVX on β_1_AR expression.^24^ Taken together, these findings indicate that oestrogen‐mediated influences on β_1_AR levels are affected by both ERα and GPR30. Furthermore, it was shown that oestrogen not only suppressed the expression of β_1_ARs but also increased sensitivity to catecholamines.^139^ Therefore, the downregulation of β_1_ARs by oestrogen may be a cardioprotective strategy against the adverse effects associated with hyperstimulation of β_1_ARs.

### 6.2 β_2_AR expression

In female rat models of I/R and heart failure, oestrogen, acting through the GPR30^24^ and ERα,^91^ increased the expression of β_2_ARs. We further showed that oestrogen in combination with testosterone enhanced the cardiac expression of β_2_ARs in OVX rats.^140^ Other researchers also reported that β_2_AR mRNA and protein were upregulated in female hearts but not male hearts in response to the arteriovenous fistula procedure.^141^ Taken together, these observations show that oestrogen decreases β_1_AR expression and upregulates β_2_AR expression in cardiac cells.

### 6.3 β_3_AR expression

Currently, information on direct effects of oestrogen on β_3_AR expression in cardiac tissues is lacking. However, variations in expression of β_3_ARs in adipose tissues have been linked to oestrogen levels. One group reported that oestrogen elevated the expression of β_3_ARs in murine adipocytes in culture,^142^ while another group observed that oestrogen decreased the quantity of β_3_ARs in brown adipose tissue of female rats in vivo.^143^ The discrepancies in these reports might be attributed to the methodologies used, that is real‐time PCR vs. radio‐ligand binding method used in the latter report or due to the inherent differences between in vitro and in vivo studies.

## 7 ERS AND βARS AS COREGULATORS OF CARDIAC CA^2+^‐HANDLING PROTEINS

Intracellular Ca^2+^ levels in cardiac cells are coregulated by both ERs and βARs. Numerous studies provide compelling evidence that oestrogen influences the expression levels of Ca^2+^‐handling proteins, whose functions are primarily under the regulation of βARs (Figure 1).^144^ In addition to the major proteins LTCC, RyR, PLB, SERCA and NCX,^27^ sarcolipin (SLN), an inhibitor of SERCA, plays a role in cardiac Ca^2+^ handling.^145^ However, to our knowledge, the influence of oestrogen on the expression or function of SLN has not been documented and hence needs to be clarified. The results of previous studies that were designed to investigate the effect of oestrogen on expression of cardiac Ca^2+^‐handling proteins channels are summarized in Table 2. In summary, the reports on oestrogen regulation of SERCA were largely consistent that oestrogen increased expression of SERCA, while its expression was decreased in OVX animal models^92^, ^93^, ^146^, ^147^, ^148^ (full reference list in Table 2). Similarly, oestrogen increased expression of NCX,^26^, ^149^, ^150^, ^151^ while it was decreased in OVX rats.^26^ However, in other studies, no change was observed in the expression of NCX expression in both oestrogen treatment and OVX animals.^152^, ^153^, ^154^ Although oestrogen decreased the expression of PLB,^155^, ^156^, ^157^ and OVX increased its expression,^155^, ^156^, ^157^, ^158^ no change in expression was found in other reports.^26^, ^147^, ^153^, ^159^, ^160^ On the other hand, oestrogen downregulated the RyR expression,^161^ while in other reports both OVX and oestrogen treatments had no effect on RyR expression.^26^, ^153^ Similarly, studies examining the role of oestrogen on the expression of LTCC yielded mixed results. Oestrogen decreased LTCC protein levels in rat ventricular myocytes (RVMs),^26^, ^161^ while OVX increased its expression in RVMs,^26^ but decreased its expression in mouse ventricular myocytes.^154^

**Table 2:** Summary of previous studies designed to investigate the effects of oestrogens on cardiac Ca^2+^‐handling proteins

Name of protein	Oestrogen effect		ER involved	Species/cell type/tissue	References
	Oestrogen	OVX			
L‐type channel	↓	↑	Not investigated	Rat ventricular tissue	26
	↑		ERα	Rabbit heart	69
	↓		Not investigated	Neonatal rat ventricular cells	161
		↓	Not investigated	Mouse ventricle tissue	154
Ryanodine receptor	─	─	Not investigated	Rat ventricular tissue	26
	↓		Not investigated	Neonatal rat ventricular cells	161
	─	─	GPR30	Rat left ventricle tissue	153
SERCA		↓	Not investigated	Rat heart tissue	158
	─	─	Not investigated	Rat ventricular tissue	26
	↑		Not investigated	Mouse apical ventricle	149
	↑		Not investigated	Zebrafish hearts	182
	─	─	ERα	Rat ventricular cells	159
	─		Not investigated	Mouse ventricular tissue	160
	↑		ERα and ERβ	Cultured murine cardiomyocytes	146
	↑	↓	Not investigated	Rat ventricular tissue	147
	↑		ERβ	Mouse ventricle tissue	92
	↑		Not investigated	Rat embryonic heart H9C2	148
	↑		ERα and ERβ	Pig coronary arteries tissue	93
	↑	↓	GPR30	Rat cardiac microsomes	162
	─	─	Not investigated	Rat heart tissue	152
	↑		Not investigated	Mouse ventricle tissue	151
	↑	↓	Not investigated	Rat heart tissue	155
	─	─	Not investigated	Rat left ventricle tissue	156
	─	─	GPR30	Rat left ventricle tissue	153
		─	Not investigated	Mouse ventricle tissue	154
	↑	↓	Not investigated	Mouse ventricle tissue	183
	↑	↓	Not investigated	Rat left ventricle tissue	157
Phospholamban		↑	Not investigated	Rat heart tissue	158
	─	─	Not investigated	Rat ventricular tissue	26
	─ Male ↑Female		Not investigated	Mouse ventricle tissue	149
	─	─	ERα	Ventricular cells	159
	─		Not investigated	Mouse ventricular tissue	160
	─	─	Not investigated	Rat ventricular tissue	147
		↓	Not investigated	Rat cardiac microsomes	162
	↓	↑	Not investigated	Rat heart tissue	155
	↓	↑	Not investigated	Rat left ventricle tissue	156
	─	─	GPR30	Rat left ventricle tissue	153
	↓	↑	Not investigated	Rat left ventricle tissue	157
NCX	↑	↓	Not investigated	Rat ventricular tissue	26
	↑		Not investigated	Mouse ventricle tissue	149
	↓		Not investigated	Neonatal rat ventricular cells	161
	↑		Genomic	Rabbit ventricular cells	150
	─	─	Not investigated	Rat heart tissue	152
	↑		Not investigated	Mouse ventricle tissue	151
	─	─	GPR30	Rat left ventricle tissue	153
		─	Not investigated	Mouse ventricle tissue	154
Sarcolipin	Not yet documented				

SERCA, sarcoplasmic reticulum Ca^2+^‐ATPase; NCX, Na^+^/Ca^2+^ exchanger pump; ER, oestrogen receptor. ↑ represents upregulation, ↓ represents downregulation and → represents no change.

These studies were carried out in different animal species, disease models, tissue/cell types, age groups, in vivo and ex vivo and using different oestrogen types. Moreover, the observed changes in protein expression due to OVX were reversed by oestrogen replacement.^26^, ^147^, ^155^, ^162^ This implies that the discrepancies between some of the results could be a result of the experimental variations, and hence, head‐to‐head comparisons might not be possible. Collectively, these findings show that oestrogen status plays a crucial role in the expression and function of cardiac Ca^2+^‐handling proteins. ERα, ERβ and GPR30 mediate these roles of oestrogen.^69^, ^92^, ^162^ Therefore, the observations that ER and βAR signalling pathways interact may have profound implications on cardiac Ca^2+^ regulation and contractility. Furthermore, through ERα,^7^ oestrogen altered myofilament Ca^2+^ sensitivity.^8^, ^154^ Indeed, in a rat model of angiotensin II‐induced hypertension, OVX exacerbated myofilament Ca^2+^ sensitivity, indicating that oestrogen deficiency may play a role in cardiac disorders by lowering the myofilament sensitivity to Ca^2+^.^8^

## 8 PHARMACOLOGICAL IMPLICATIONS AND THERAPEUTIC OPPORTUNITIES

### 8.1 Effects of the interactions on drugs targeting βARs and Ca^2+^ channel blockers

Interactions between ER and βAR pathways could have broad implications in the clinical context. β‐Blockers and Ca^2+^ channel blockers are 2 mainstays for the treatment of cardiovascular disease.^163^ These drugs control the heart rate and blood pressure by modulating the activation of βARs. However, there are conflicting observations regarding their effectiveness in managing conditions such as hypertension.^163^ Reports from cohort studies have noted that some patients, particularly women, under β‐blockers are unable to reach targeted blood pressure compared to men.^164^ This observation can be explained, partially, by the aforementioned influence of oestrogen on βARs' function. Moreover, gender and age differences in expression of β_1_ARs/β_2_ARs have been reported, which may be attributed to oestrogen.^135^ Besides, gender variations in responses to catecholamines,^139^ and in cardiac Ca^2+^ handling,^165^ have been observed in animal experiments. Therefore, the efficacy of β‐blockers and Ca^2+^ blockers may vary with gender or age groups based on the interplay between ERs and βARs. As demonstrated, carvedilol, a non‐selective β‐blocker, protected against myocardial contractile dysfunction caused by oestrogen deficiency.^158^ Interestingly, this is one of the β‐blockers to display biased agonism and it too can activate the β_2_AR‐G_αi_/β‐arrestin pathways.^166^, ^167^ With the current understanding of the ER and βAR pathways, further studies should examine how the efficacy of the drugs targeting these receptors and/or their signalling pathways may be altered in the context of the ER and βAR crosstalk. Theoretically, oestrogen by inhibiting the LTCC or altering the expression of βARs might indirectly compromise the functions of Ca^2+^ blockers and β‐blockers respectively. Consequently, men and women may respond differently to these classes of drugs. The crosstalk may inform the decisions regarding the choice of antihypertensive drugs to patients with consideration to age and gender.

### 8.2 Therapeutic opportunities

The functional synergism between ERs and βARs provides therapeutic avenues for cardioprotection. For example, while β_1_AR activation promotes CaMKII‐induced apoptosis,^25^, ^112^ β_2_AR, ERα and GPR30 activation seems to act in a manner that promotes anti‐apoptosis through the G_i_/PI3K/Akt pathway. Considerations for strategies targeting the PI3K/Akt pathway will provide a feasible avenue for cardioprotection. For instance, the ability of ERα binding to the alpha subunit of PI3K seems attractive, as it is more specific and avoids various points of integration between ER and βAR signalling cascades discussed above. Activation of Akt pathway will protect against mitochondria‐associated apoptosis induction. Moreover, Akt was shown to act as a surrogate molecular ligand for ERα that induced expression of oestrogen‐regulated cardioprotective genes in breast cancer cells.^168^ This perspective partly indicates that selective activation of this pathway would potentially enhance the cardiac functions under pathological conditions. Another therapeutic target is the possibility of direct binding of oestrogen to the LTCC. Oestrogen‐LTCC docking studies may help to predict the mode of binding/interaction of this complex. This approach will advance the prospects of oestrogen as a Ca^2+^ channel blocker if appropriate technologies are applied to enhance its specificity. We anticipate that the therapeutic value of this manoeuvre could be a potential target for Ca^2+^‐related pathologies such as arrhythmia treatments.

## 9 CONCLUSION

This review demonstrates the expression patterns and functions of ERs and βARs in cardiovascular tissues. The data sets reviewed above show some inconsistencies with regard to tissue‐specific expression of the ERs. For instance, it is not clear whether ERβ is expressed in adult cardiomyocytes, and the cellular localization of GPR30 is not clear. Therefore, further studies are warranted to resolve these observations. In addition, the reviewed data sets strongly support the hypothesis that ERs and βARs function as collaborative partners in modulating the physiology of the cardiovascular system. The recently described oestrogen receptor GPR30 mimics the dual coupling of the β_2_ARs to the G_αs_ and G_αi_ proteins. On this basis, oestrogen pathways play into the network of the βAR signalling cascades. Furthermore, GPR30 and βARs show similarities with regard to their ability to associate with AKAPs, PDZ motif‐binding proteins and possession of PKA and CaMKII binding sites. Despite the signalling pathways discussed in this review, the functions of the GPR30 and other ERs remain incompletely understood. Further research is required to uncover the identities of signalling molecules that orchestrate their functions.

The crosstalk between the ERs and βARs could have implications on drugs that target these receptors, especially β‐blockers and Ca^2+^ channel blockers. Oestrogen influences the expression of βARs and Ca^2+^‐handling proteins, which could compromise the efficacy of the drugs in a gender‐dependent manner. This perspective requires further evaluation at the clinical level. In addition, the concept of direct oestrogen binding to the LTCC might be of great clinical relevance as oestrogen might be used to design Ca^2+^ channel blockers. This opens a window of research into the receptor‐independent pathways for oestrogen. Furthermore, future research should exploit the therapeutic potential of the cardioprotective PI3K/Akt pathway that is activated downstream of both ERs and βARs.

## CONFLICT OF INTEREST

The authors report no conflict of interests.
